# A review of patient-reported outcomes used for regulatory approval of oncology medicinal products in the European Union between 2017 and 2020

**DOI:** 10.3389/fmed.2022.968272

**Published:** 2022-08-12

**Authors:** Maria Manuel Teixeira, Fábio Cardoso Borges, Paula Sousa Ferreira, João Rocha, Bruno Sepodes, Carla Torre

**Affiliations:** ^1^Faculdade de Farmácia da Universidade de Lisboa, Lisbon, Portugal; ^2^European Organization for Research and Treatment of Cancer (EORTC), Brussels, Belgium; ^3^Laboratory of Systems Integration Pharmacology, Clinical and Regulatory Science, Research Institute for Medicines (iMED.ULisboa), Lisbon, Portugal

**Keywords:** oncology, patient-reported outcome (PRO), patient-reported outcome measures (PROMs), European Medicines Agency (EMA), summary of product characteristics (SmPC) claims

## Abstract

**Introduction:**

Cancer and corresponding available treatments are associated with substantial symptoms and functional limitations. In this context, collection of patient-reported outcomes (PRO) in clinical trials gained special interest and is recommended by regulatory authorities. Within clinical trials framework, PRO may provide evidence to support medicines approval, labeling and marketing claims. This study aims to analyze the existing evidence based on PRO as part of new oncology indications receiving positive opinions issued by the European Medicines Agency (EMA) between 2017 and 2020 and to identify PRO related label claims granted.

**Methodology:**

Oncology medicinal products and indications approved by the European Commission following a positive opinion from the EMA between 2017 and 2020 were identified. European Public Assessment Report (EPAR) and Summary of Product Characteristics (SmPC) were reviewed for each medicinal product to identify use of PRO and PRO label claims.

**Results:**

A total of 128 oncology indications, corresponding to 76 medicines, were approved; of those, 100 (78.1%) included PRO in the confirmatory clinical trials. Thirty-seven indications were supported by double-blind randomized trials and the remainder 63 by open-label trials. Out of the 104 confirmatory trials analyzed, PRO were defined as a secondary endpoint in 60 studies (57.7%), exploratory in 31 (29.8%) and as both in 13 (12.5%). In total, 54 different PRO measures (PROM) were used, of those 41 (75.9%) were disease-specific measures. Nevertheless, PROM selected relied on the EORTC (41.3%), FACIT (17.1%) and EQ-5D (29.2%) measures. A total of 76 indications (59.4%) had PRO reviewers comments included in the EPAR, however only 22 indications (17.8%) included label claims in the SmPC. The reasons identified in the EMA assessment supporting the exclusion of PRO claims were described for 34 indications (44.7%).

**Conclusions:**

Despite growing recognition of the value of PRO data for the development of improved cancer therapies, PRO implementation remains challenging. The main reasons identified in our study are related with study design, missing data, study conduct and PROM selection.

## Introduction

Capturing patient's perspective during clinical trials in the oncology setting is an opportunity to collect unique information on the patient's experience of the disease, its treatment and, most importantly, the impact on their quality of life, which may contribute to develop more appropriate health care interventions (1–3). Patient-Reported Outcomes (PRO) provide a holistic approach of treatment effects, and have been increasingly recognized as an essential complement to clinical [e.g., overall survival (OS), progression-free survival (PFS) and safety] and laboratory-related (e.g., biomarker) outcomes (4). The use of PRO measures (PROM) is encouraged both by the European Medicines Agency (EMA) and the Food and Drug Administration (FDA) guidelines for anticancer medicinal products development (1), covering single and multi-dimension measures such as symptoms, feelings, functioning, well-being and treatment satisfaction (5–7). Inclusion of PRO as an endpoint in a clinical trial is of added value for various stakeholders beyond regulators, including the health technology assessment (HTA) bodies/payers and professional organizations, as they contribute to distinct among products with similar survival benefits (1–3, 6–9).

The instruments developed to collect valid and reliable PRO are designated as PROM and may be classified as generic or disease-specific (10, 11). Generic instruments are designed to measure health concepts and, although these might lack sensitivity about important disease domains, are valid for a variety of patient populations, allowing across-disease comparisons (6, 9–13). Conversely, disease-specific instruments are designed to measure health outcomes and tend to have higher clinical relevance and change responsive (10–13). However, they may not be able to capture unexpected treatment-related toxicities, do not allow comparisons between conditions and, unlike generic PROM, they are not easily incorporated in economic evaluations (6, 9, 10, 12, 13).

It is acknowledged that cancer diseases and respective treatment regimens, are associated with both substantial symptoms and functional limitations. In this sense, patients' perspective into all aspects of cancer care treatment, especially regarding benefits and risks of a treatment, are of added value (14–17). PRO have increasingly been used within oncology clinical trials to assess domains of patients' health status without the introduction of third-party bias. These analyses are of special interest in oncology clinical trials, where quality of life might be more important than longevity (7, 14).

Even though there is no consensus on which PRO are appropriate to select as clinical trial endpoints, they should assess a specific concept, such as a symptom, function, or Health Related Quality of Life (HRQoL) (10, 18). Within the clinical trials framework, PRO may provide evidence supporting medicines approval, labeling and marketing claims (10, 19, 20). Despite early recognition by regulatory authorities of the importance of PRO use in oncology, this poses several challenges, namely: (i) lack of standards in the selection and methodology of PRO used; (ii) poor conduct of studies resulting in missing data and bias; (iii) misleading side effects with disease symptoms; (iv) existent measures failing to capture the concerns of asymptomatic patients and; (v) the increasing use of open-label or single-arm designs. Additionally, regulatory authorities differ in the criteria for inclusion of PRO data in labeling (21). Nevertheless, the integration of PRO into regulatory decision-making process is of growing interest and regulatory agencies have collaborated internationally to harmonize PRO incorporation, e.g., contributing to the EORTC's Setting International Standards in Analyzing Patient Reported Outcomes and Quality of Life Endpoints Data (SISAQOL) initiative, to harmonize recommendations for the analysis of PRO data from clinical trials; or to the SPIRIT-PRO guidelines aiming to standardize PRO item protocols (21–23).

The use of PRO into confirmatory oncology clinical trials has been increasing over the last two decades, and have demonstrated to be predictors of OS (3, 6, 24). In a study that reviewed PRO labeling for oncology medicinal products approved by the FDA and EMA between 2012 and 2016, it was stated that 70.3% of approved oncology indications included PRO data. Furthermore, it was also reported that both agencies identified the missing PRO data as problematic for data. The most frequently used PROM were EORTC and FACIT measures. These questionnaires assess common cancer symptoms and toxicity, including selected frequent symptomatic adverse effects of cancer (e.g., fatigue, vomiting, diarrhea) and pain. The variety of new oncology medicines in the last few years presents a different toxicity profile, making the use of these static tools questionable (1). Thus, it is of utmost relevance to characterize the use of PRO in recent years and to understand whether its utilization is increasing and better supporting regulatory decisions. In this context, the aim of this study was to analyse the data based on PROs of new oncology indications granted a market authorization by the European Commission following a positive opinion by EMA between 2017 and 2020, in order to further substantiate and update the current understanding and available evidence.

## Methods

This is a descriptive study. New oncology indications, including line extensions, granted a market authorization by the European Commission following a positive opinion by EMA between 2017 to 2020 were identified through a systematic comprehensive electronic and manual search of the Committee on the Medicinal Products for Human Use (CHMP) meeting minutes (25). Medicinal products with one or more indications approved within study timeframe were included. Medicinal products withdrawn at the time of data extraction, Medicinal products with only modifications to the existing labeling and with no new indications granted were excluded. The identification and selection of the medicinal products to be included in the study were performed by two independent investigators. Disagreements over medicinal products selection phase were discussed among the two investigators to determine the final list of medicines and the correspondent therapeutic indications included in this review.

For the purposes of this analysis, medicines were classified by cancer type, according to the classification provided on the EMA website (26). For each medicine and correspondent active substance(s), the European public assessment report (EPAR) and the summary of product characteristics (SmPC) were retrieved from EMA website (26). An EPAR is a resource document published by EMA to describe how the medicine was assessed, including information on the design and conduct of clinical trials; a SmPC is a legal document approved as part of a marketing authorization that provides healthcare professionals with information on how to use the medicine, including results of the clinical trials used to support approval.

Data extraction from EPARs was performed by one investigator and independently verified by other two investigators. Disagreements were resolved resorting to a consensus strategy, based on joint decision-making. The following data were extracted: (a) brand name and international non-proprietary name (INN); (b) therapeutic indication; (c) marketing authorization holder; (d) main study(ies) supporting submission, (e) study design; (f) comparator(s); (g) number of patients included; (h) use of PRO and PROM; (i) PRO endpoint status (primary, secondary and/or exploratory); (j) PROM designation; and (k) EPAR assessment comments.

Only main studies, e.g., confirmatory trials, were included in the analysis, i.e., supporting studies were not considered. Medicines for which approval was based on multiple main studies, all studies were included in the analysis. EPAR assessment comments were extracted to potential identify causes for inclusion and/or exclusion of SmPC label claims. Moreover, both CHMP Summary of Opinion date and the European Commission decision issued date were identified.

PROM used were categorized as generic and disease-specific and its selection was assessed. Furthermore, the section 5.1 *Pharmacodynamic Properties* of the screened SmPC was reviewed for PRO label claims. For analytical purposes, the identified PRO claims were classified as HRQoL, global health status, symptoms and/or functioning related.

## Results

### Characterization of the studies that included PRO

**Figure 1:**
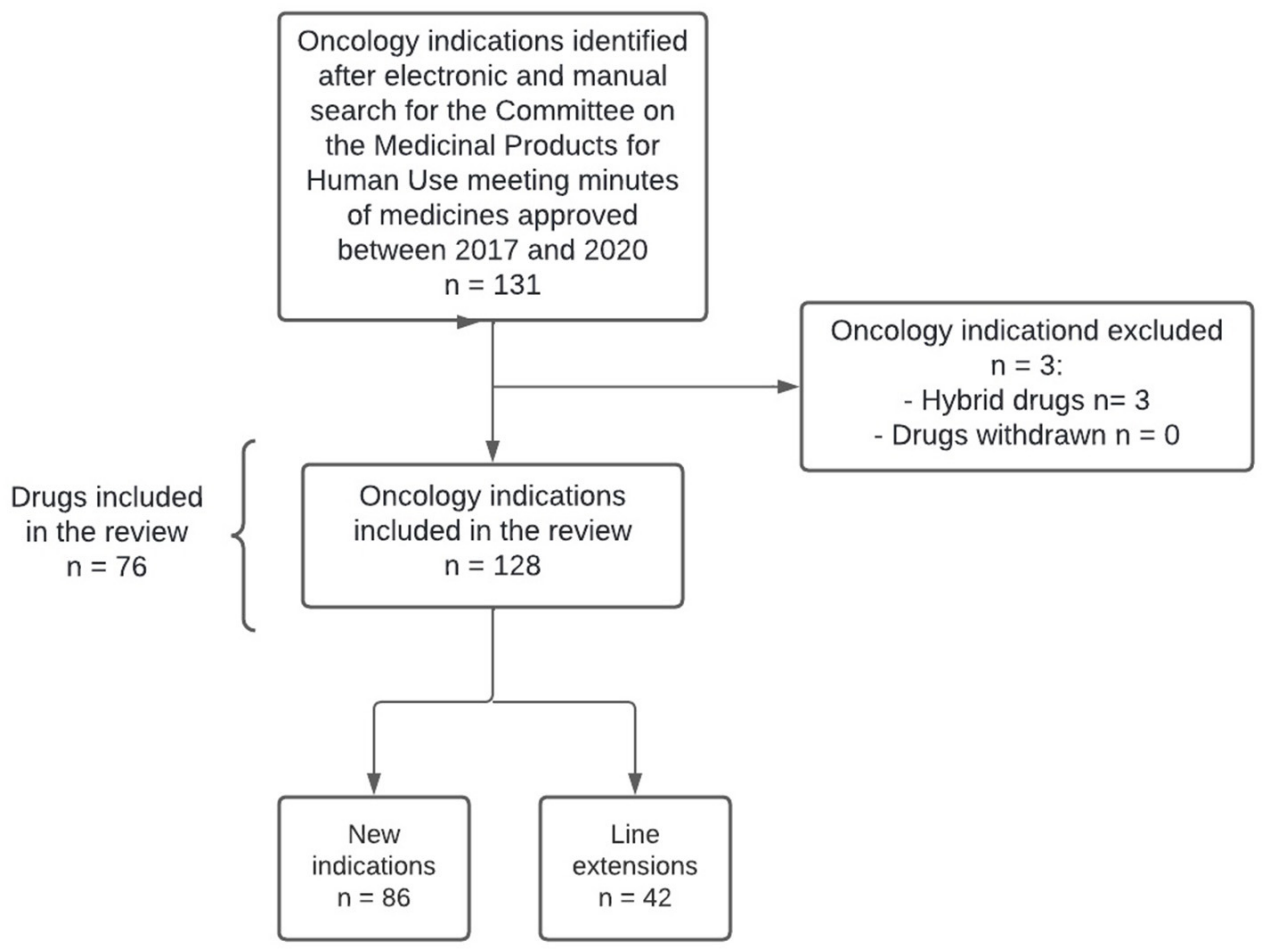
Research methodology flowchart.

A total of 132 oncology indications were first identified and four were subsequently excluded, resulting in a total of 128 oncology indications included (86 new indications and 42 line extensions), corresponding to 76 medicines (Figure 1). Among the 76 medicines approved, 28 (36.8%) received two or more approvals for oncology indications (Supplementary materials - Annex 1).

Out of the 128 oncology indications approved, 100 (78.1%) included PROM in the confirmatory studies supporting the submission, of which the majority corresponded to solid tumor indications (72.0%). Among the 42 line extensions approved, 21 (50%) included PROM in the confirmatory studies. Overall, only 22 indications (17.2%) included label claims in the SmPC (Table 1).

**Table 1:** Summary of patient-reported outcomes and patient-reported outcomes label claims.

**Cancer type**	**Number of indications reviewed**	**Number of indications with patient-reported outcomes *n* (%)**	**Number of indications with patient-reported outcomes labeling *n* (%)**
**Solid tumors**			
Abdominal neoplasm	1	1 (100%)	0 (0%)
Adenocarcinoma	1	1 (100%)	0 (0%)
Basal cell carcinoma	1	0 (0%)	0 (0%)
Breast neoplasm	12	11 (91.7%)	4 (33.3%)
Colorectal neoplasms	1	1 (100%)	0 (0%)
Gastrointestinal neoplasm	2	1 (50%)	0 (0%)
Hepatocellular carcinoma	5	5 (100%)	1 (20%)
Melanoma	8	7 (87.5%)	3 (37.5%)
Merkel cell carcinoma	1	1 (100%)	0 (0%)
Neuroblastoma	1	0 (0%)	0 (0%)
Non-small-cell lung carcinoma	20	16 (80%)	5 (25%)
Ovarian neoplasm	8	7 (87.5%)	1 (12.5%)
Pancreatic neoplasm	1	1 (100%)	1 (100%)
Prostatic neoplasms	8	7 (87.5%)	2 (25%)
Renal cell carcinoma	6	4 (66.7%)	0 (0%)
Small-cell-lung carcinoma	1	1 (100%)	0 (0%)
Solid tumors	1	1 (100%)	0 (0%)
Squamous cell carcinoma	4	3 (75%)	0 (0%)
Urologic neoplasms	3	3 (100%)	1 (33.3%)
**All solid tumors**	85	72 (84.7%)	18 (21.2%)
**Hematological malignancies**			
Acute lymphoblastic/ lymphocytic leukemia	5	2 (40%)	1 (20%)
Acute myeloid/myeloblastic leukemia	5	1 (20%)	0 (0%)
B-cell lymphoma	4	2 (50%)	0 (0%)
Chronic lymphocytic leukemia	6	6 (100%)	0 (0%)
Chronic myeloid meukaemia	2	1 (50%)	0 (0%)
Follicular lymphoma	2	2 (100%)	1 (50%)
Hodgkin lymphoma	2	2 (100%)	1 (50%)
Mantle cell lymphoma	1	1 (100%)	0 (0%)
Mastocytosis	1	1 (100%)	0 (0%)
Multiple myeloma	11	7 (63.6%)	0 (0%)
T-cell lymphoma	3	3 (100%)	1 (33.3%)
Waldenström's macroglobulinemia	1	1 (100%)	0 (0%)
**All hematological malignancies**	43	28 (65.1%)	4 (9.3%)
**Total**	128	100 (78.1%)	22 (17.2%)

Regarding the 100 indications with PRO data (corresponding to a total of 104 clinical trials), 37 (37.0%) were supported by double-blinded randomized controlled trials (RCT) and the remainder 63 were supported by open-label trials: randomized or single arm trials. A total of 15 (14.4%) clinical trials enrolled <200 patients: seven clinical trials included 50 to 99 subjects, five included 100 to 150 subjects, and three included 149 to 200 subjects. Out of the 104 clinical trials including PRO, these were defined as a secondary endpoint in 60 (57.7%) studies. A total of 31 studies (29.8%) included PRO as an exploratory endpoint and 13 studies (12.5%) included PRO both as secondary and exploratory endpoints. The studies comprising PRO data are further characterized in Table 2.

**Table 2:** Characterization of the studies that included patient-reported outcomes (*n* = 104).

**Characteristics**	**Solid tumors *n* (%)**	**Hematological malignancys *n* (%)**
Double-blind RCT	36 (34.6%)	3 (2.9%)
Open-label RCT	30 (28.8%)	21 (20.2%)
Open-label single arm	9 (8.7%)	5 (4.8%)
<200 patients enrolled	7 (6.7%)	8 (7.7%)
Endpoint Status Secondary Exploratory Secondary + Exploratory	74 (71.2%) 45 (43.3%) 21 (20.2%) 8 (7.7%)	30 (28.8%) 15 (14.4%) 10 (9.6%) 5 (4.8%)

### PROM selected

A total of 54 different measures were used, within which 41 (75.9%) were disease-specific (Supplementary materials - Annex 2). Noteworthy, 50 PROM were rarely used, i.e,. for <10 studies, suggesting wide variability in the measures selected. The majority of the trials (82.7%) used more than one PROM (a total of 240 PROM were used across the 100 indications with PRO data).

PRO assessments relied most frequently on the European Organization for Research and Treatment of Cancer (EORTC) (41.3%), Functional Assessment of Chronic Illness Therapy (FACIT) (17.1%) and Euroqol-5 Dimension (EQ-5D) (29.2%) measures (Table 3). FACT-G was supplemented with a FACIT module in about 60% of the trials, while the EORTC QLQ-C30 was supplemented in about 50% of the times selected.

**Table 3:** Patient-reported outcome measures selected for the studies included in the review.

**Patient-reported outcome measure**	**Number of times used *n* (%)**
Euroqol-5 Dimension Index	70 (29.2%)
Other Generic measures	18 (7.5%)
European Organization for Research and Treatment of Cancer (EORTC) modules EORTC QLQ-C30 with EORTC disease-specific module EORTC QLQ-C30 without EORTC disease-specific module	99 (41.3%) 32 (32.3%) 30 (30.3%)
Functional Assessment of Chronic Illness Therapy (FACIT) measures FACT-G with FACT disease-specific measure FACT-G without FACT disease-specific measure	41 (17.1%) 3 (7.3%) 2 (4.9%)
Other disease-specific measures	12 (5%)
**Total**	240 (100%)

Overall, non-small cell lung cancer (NSCLC) and breast cancer were the cancer types with more medicines including PROM in the confirmatory trials, with 16 (80% of the NSCLC studies) and 11 (91.7% of the breast cancer studies), respectively. These were followed by seven medicines in melanoma, ovarian and prostatic neoplasms (corresponding to 87.5% of the studies in each cancer type), and multiple myeloma (63.6% of the multiple myeloma studies).

### EMA reviewers' commentaries and SmPC claims

Out of the 128 approved oncology indications, 76 (59.4%) had EMA reviewers commentaries on PRO included in the EPAR. EMA reviewers' comments provided possible reasons for not including PRO data in the SmPC for 34 indications (44.7%). The comments focused mainly on study conduct, study design, PROM selection and missing data. The most recurrent argument pertained to the caution needed for PRO data interpretation when using an open-label design. For a total of 20 indications, no reason for claim exclusion was identified. The EMA reviewers noted that quality of life data would be of interest on 2 indications, corresponding to axicabtagene ciloleucel and glasdegib, but were not presented. The reasons for PRO label claims exclusion identified in EPAR are presented in the Table 4 (it was considered that a comment may include one or more reasons for PRO label exclusion).

**Table 4:** Reasons for patient-reported outcomes label claims exclusion identified in European public assessment reports reviewers ' comments (*n* = 76).

**Comments from EMA reviewers**	**Number of indications *n* (%)**
**Study conduct**	
Data should be interpreted with caution as there was no blinding of the study treatment	1 (1.3%)
Potential bias in PRO data as a result of blinding failure	2 (2.6%)
Interpretability of QoL results and therefore their clinical relevance is unclear/limited	8 (6.6%)
Rational for timing and frequency of PRO collection was not fully described with regard to population, disease and/or treatment regimen	4 (5.3%)
PRO analysis was not robust enough or did not even exist	4 (5.3%)
PRO analysis was considered exploratory	4 (5.3%)
**PROM selection**	
PROM selected was not considered optimal	4 (5.3%)
**Missing data**	
Handling missing data was not included and/or sufficient	2 (2.6%)
Reliability of the results was hampered due to missing data	5 (6.6%)
**Study design**	
Value of data was questionable and caution in interpretation is needed when using open-label design	16 (21.1%)
No firm conclusion could be drawn from the QoL data of single arm trials	2 (2.6%)

Label claims were granted for only 22 (17.2%) therapeutic indications approved within study timeframe, the majority corresponding to solid tumors. Only 18.2% of the indications with PRO labeling corresponded to hematological malignancies. Table 5 presents the types of claims granted by EMA and the PROM associated; Supplementary materials - Annex 3 provides detailed information extracted from the SmPC claims. Among the oncology indications with SmPC claims, 11 (50%) were supported by randomized open-label studies, 10 (45.5%) by double-blind RCT and 1 (4.5%) by an open-label single arm study. PRO was selected as a secondary endpoint in 16 studies (72.7%), as exploratory in 4 (18.2%) and as both secondary and exploratory in 2 (9.1%). Among the studies with SmPC claims granted, only one enrolled <200 patients.

**Table 5:** Types of claims granted.

**Cancer type**	**Medicine generic name**	**PROM**	**Type of claim granted**	**Study design**
ALL	Tisagenlecleucel	PedsQL EQ-5D	HRQoL	Open-label single arm trial
Breast Neoplasms	Abemaciclib	BPI EORTC QLQ-C30 EORTC QLQ-BR23 EQ-5D 5L	HRQoL (no difference between groups)	Double-blind RCT
Breast Neoplasms	Fulvestrant	EORTC QLQ-C30 EORTC QLQC30 EQ 5D	Symptoms	Double-blind RCT
Breast Neoplasms	Pertuzumab	EORTC QLQ-C30 EORTC QLQ-BR23 EQ-5D	Global health status, symptoms, functioning	Double-blind RCT
Breast Neoplasms	Ribociclib	EORTC QLQ-C30 EORTC QLQ-BR23 EQ-5D-5L	HRQoL, global health status (no difference between groups)	Double-blind RCT
FL	Obinutuzumab	FACT-Lym EQ-5D-3L	Symptoms HRQoL and global health status (no difference between groups)	Open-label RCT
HCC	Cabozantinib	EQ-5D-5L	HRQoL	Double-blind RCT
HL	Brentuximab Vedotin	EORTC QLQ C30 FACT/GOG-Ntx subscale EQ-5D-3L	HRQoL (no difference between groups)	Open-label RCT
Melanoma	Binimetinib	FACT-M EQ-5D-5L EORTC-QLQ-C30	HRQoL, functioning, symptoms	Open-label RCT
Melanoma	Encorafenib	FACT-M EQ-5D-5L EORTC QLQ-C30	HRQoL, functioning, symptoms	Open-label RCT
Melanoma	Nivolumab	EORTC QLQ-C30 EQ-5D WPAI:GH	HRQoL (no difference between groups)	Double-blind RCT
NSCLC	Atezolizumab	EORTC QLQ-LC13	Symptoms	Open-label RCT
NSCLC	Atezolizumab	EORTC QLQ-C30 EORTC QLQ-LC13 SILC scale	HRQoL, global helath status (no difference between groups)	Open-label RCT
NSCLC	Ceritinib	EORTC QLQ-C30 EORTC QLQ-LC13 LCSS EQ-5D	HRQoL, global helath status, symptoms	Open-label RCT
NSCLC	Durvalumab	EORTC QLQ-C30 EORTC QLQ-LC13 WHO PS Scores	HRQoL, functioning, symptoms (no difference between groups)	Double-blind RCT
NSCLC	Osimertinib	EORTC QLQ-C30 EORTC QLQ-LC13	HRQoL,global helath status, functioning, symptoms (no difference between groups)	Double-blind RCT
Ovarian Neoplasms	Niraparib	FOSI EQ-5D-5L Neuropathy Questionnaire	HRQoL (no difference between groups)	Double-blind RCT
Pancreatic Neoplasms	Lutetium (177Lu) oxodotreotide	EORTC QLQ-30 EORTC G.I.NET21	HRQoL	Open-label RCT
Prostatic Neoplasms	Apalutamide	FACT-P EQ-5D	HRQoL (no difference between groups)	Double-blind RCT
Prostatic Neoplasms	Padeliporfin	IPSS IIEF-15 EQ-5D	Symptoms	Open-label RCT
T-cell Lymphoma	Brentuximab vedotin	Skindex-29 questionnaire FACT-G EQ-5D-3L	HRQoL (no difference between groups)	Open-label RCT
UC	Pembrolizumab	EORTC QLQ-C30 EUROQoL EQ-5D	HRQoL, global health status	Open-label RCT

## Discussion

The use of PROM provides clear benefits for both oncology research and clinical practice (23, 27). In clinical research, PRO are particularly important as they complement clinical endpoints and, by including patients' experience, allow for a better adverse event characterization (28). However, despite regulatory developments and increasing emphasis on the use of PRO, the influence of patients' perspective on oncology medicinal products approval decisions remains challenging. This was highlighted in our study, as between 2017 and 2020, EMA granted PRO labeling to only 22 (17.2%) out of the 128 oncology indications approved during this period. This is even lower than what was observed in a previous review of PRO labeling for oncology medicinal products approved between 2012 and 2016, where EMA granted PRO labeling to 21 (32.8%) out of the 64 indications approved (1, 29, 30). It is worth mentioning that in our study 78% of the approved indications include PRO while in the previous review PRO were included in 70% of the indications, thus suggesting an increase in PRO use.

An important concern when including PRO in clinical research is methodological robustness and consistency of outcome reporting. General barriers regarding PRO inclusion in clinical trials are well-identified in the literature, such as uncertainties in choosing the PROM and timing for data collection, difficulty in designing statistical analysis and interpreting results, as well as misalignment criteria with regulatory agencies (1, 23, 27, 28). The main reasons identified in our study for poor inclusion of the patient perspective were related to study design, missing data, study conduct and PROM selection.

### Study design

Our study results showed that 11 (50%) trials with PRO SmPC claims granted by EMA between 2017 and 2020 were supported by open-label trials, 10 (45.5%) were supported by double-blind RCT and 1 (4.5%) was supported by an open-label single arm study. Furthermore, a total of 51 out of the 104 trials with PRO data were open-label RCT, of which 19 had an EMA assessment comment stating than open-label design should be taken into consideration when interpreting results. This is consistent with EMA guidance, which recognize that PRO results from open-label designs can be considered when supported by prior and thorough planning (6).

Study design has been consistently identified as a key concern (1, 28, 29). Both EMA and FDA recommend double-blind designs for an accurate PRO collection, since it is considered that open-label designs are rarely adequate to support label claims, as they might introduce observer bias (6, 19, 31–36). Regulatory authorities' arguments are that patients' responses may be influenced if they are aware of their treatment allocation, with potential disappointment if enrolled in the control arm, or satisfaction if assigned to the experimental arm, possibly contributing to both study dropouts and missing data in the control arm (28, 32, 34–37). Nonetheless, even though more thorough and comprehensive analysis are needed, such concerns have been deconstructed (28, 34, 38, 39).

Two reviews conducted by Roydhouse et al. (34, 38) investigated the potential bias in PRO in open-label cancer trials by comparing PRO response rates among study arms in double-blind and open-label cancer clinical trials submitted to FDA between 2007 and 2017. Although response rates were high and comparable across designs, large differences (more than 10%) between arms were found in both designs, 26.9% for double-blind trials and 16% for open-label trials. Despite differences favoring experimental arm being more frequently found in open-label trials, PRO results were not systematically favoring open-label RCTs. Thus, current evidence does not support the concerns previously expressed. Although challenging, one should start considering PRO data in open-label and single-arm trials (SAT), while supporting research to fully characterize un-blinding effects on PRO results (21, 28, 39).

A possible strategy to mitigate PRO results bias in open-label arms is to compare baseline measures before and after randomization and/or treatment. In addition, the possibility of completion and/or dropout bias should be mitigated through PRO completion reports, patient enrolment in both arms across the trial and by planning sensitivity analyses to test PRO results robustness (28, 34, 38). As for concerns about the interpretation of PRO results retrieved from SAT, the recommendation could be resorting to pre-specified thresholds for a significant score change in the concepts of interest, or to assess only symptomatic adverse effects that foster understanding on therapy tolerability (21, 40).

### Missing data

In our study, corroborating evidence observed was aligned with previous reviews; missing PRO data has been criticized by EMA reviewers for seven indications (9.2%), as it might affect analysis and interpretation of PRO results. Consequently, no PRO label claims were granted for the corresponding indications. Additionally, two reviewers' comments on two indications with open label clinical trials, mentioned that the results should be interpreted with caution due to the small sample size.

Since the number of participants decreasing throughout a cancer clinical trial is a reality, missing PRO data is a common concern, so the nature and extent of the missing data should be considered when interpreting PRO results (1, 32, 41–45). PRO missing data may lead to biased results, decreasing the internal and external validity of the trial, decreasing the power of the trial to detect differences between arms, and ultimately waste resources (42).

A systematic review of 33 RCTs, performed by Hamel et al. on the quality of statistical methods to analyse QoL data in cancer trials, concluded that PRO compliance rates ranged between 45 and 90%. None of the RCTs included in the review provided complete HRQoL data on all the planned measurement times and only 6 implemented an analysis procedure for PRO data (41). Other recent review conducted by Palmer and colleagues identified 46 factors for PRO missing data in the literature and categorized them on five main components: instrument, participants, center, staff, and study. However, the importance attributed for each risk factor is unknown, as the strength of evidence supporting each one was variable (42, 45). Additionally, small patient numbers in studies may affect both study design robustness and statistical methodology, so that PRO results retrieved from such small studies are more likely to be biased by missing data (21).

Clearly, PRO missing data is a substantial and complex problem which has been historically associated with sponsors and investigators commitment, and strategies to prevent it need to be implemented (1, 21, 41, 42). An identified methodology to address this concern is by defining *a priori* statistical analysis techniques. However, missing data due to administrative errors can be difficult to address in a sensitive analysis. Therefore, it is acknowledged that improving the design and conduct of the studies is considered the most appropriate approach toward preventing PRO missing data. As such, several strategies have been proposed: incorporate PRO timings assessments in the protocol, reduce patient burden in PROM completion, select staff to coordinate and monitor PRO activities throughout the study, and continually educate sponsors, investigators and patients on PRO completion importance (21, 42, 46, 47). Our findings fully support the strategies proposed, as EMA assessment comments identify both the absence or insufficient description for timing and frequency of PRO collection, and the absence or insufficiently robust analysis of PRO data as problematic.

### PRO data

In this study we concluded that, consistently to what was demonstrated in previous oncology clinical trials, the most frequently PROM used was the generic measure EQ-5D and the disease-specific EORTC and FACIT measures (1, 45, 48). Additionally, we observed that FACIT measures, although used less frequently, supported a slightly higher proportion of PRO label claims (17.1%), when compared to EORTC measures (15.2%). This is consistent with the results from the review of PRO labeling for oncology medicinal products approved by the FDA and the EMA between 2012 and 2016, which deconstructed the belief that EMA prefers data based on EORTC modules (1, 49).

#### PROM selection

The selection of a PROM should reflect the purpose and objectives of the study, as well as the QoL domains most appropriate for the target population and endpoints being assessed (6).

The EQ-5D is a generic instrument and was developed to measure, compare, and assess health status across disease areas. Nowadays is used to measure patients' health status, but also is extensively used to support HTA cost-effectiveness studies (50, 51). We were able to conclude that EQ-5D was used in 63% of the studies with PRO data. This is consistent with the results of a systematic review assessing PROM selection in cancer trials conducted between 2004 and 2019, which acknowledged a clear increase in the EQ-5D use attributable to the relevance of this instrument with HTA and reimbursement purposes (48, 51).

EORTC QLQ and FACIT measures focus on HRQoL assessment and they both consist in a cancer-specific core measure, the EORTC QLQ-C30 and FACT-G, respectively (45, 48). We observed that FACT-G was supplemented with a FACIT module in 60% of the trials, while the EORTC QLQ-C30 was supplemented in about 50% of the times selected. This discrepancy is consistent with the results of the systematic review assessing PROM selection in cancer trials (2004–2019) previously mentioned, which may reflect that EORTC QLQ-C30 includes domains for assessing major cancer symptoms in addition to functional health domains, whereas the FACT-G focuses on functional health with symptoms being assessed within its disease-specific modules (48). Although the EORTC QLQ and FACIT measures continue to be the most widely PROM used in cancer trials, their use irrespectively of disease stage or cancer type is questionable. Given these are measures with a focus on a broad concept, i.e., HRQoL, they may lack sensitivity, meaning that some items may be irrelevant and at the same time, may not sufficiently capture patients' experiences with a particular therapy (1, 6, 19, 49). In a previous study which evaluated PRO data from 18 immunotherapy studies it was observed that immune-related adverse events were not consistently assessed, suggesting that such measures may not be appropriate for new approved therapies (52). In our study, 30 of the 54 measures selected are cancer-specific, supporting the need to develop more appropriate measures. Developing novel PROM is becoming challenging with the rapid and constant evolution of cancer care. Although several PROM and extensive literatures exist, selecting the most appropriate, and validated, questionnaire remains a challenge, hence there is a need to develop methods to combine or improve existing measures in a thoughtful way (21).

#### PRO label claims

Our study showed that only 17.2% of the indications reviewed included PRO claims in the SmPC. About half of these claims were based on results with no clinical significance between arms. EMA PRO label claims granted between 2017 and 2020 relied on PRO as a secondary endpoint in 72.7%, as exploratory in 18.2% and as both secondary and exploratory in 9.1%. Additionally, label claims mentioned broad concepts such HRQoL and global health status in 86% of the claims, while 45% mentioned symptoms and only 23% functioning. Such results are in line with previous reviews, which concluded that EMA more often approves QoL claims, whilst the FDA approves more frequently symptom-related claims (21, 53). As noted by the authors in the review of PRO label claims by the EMA and FDA between 2012 and 2016, there is no uniform definition in the concepts of HRQoL or global health status in the label granted by EMA (1). As these concepts can be influenced by several factors such as age, culture, comorbidities, etc., the FDA does not consider them to be reliable for labeling purposes. As *per* both EMA PRO guidance, EMA considers that HRQoL goes beyond efficacy and safety evaluations. Therefore, SmPC claims will always be considered, as they are recognized as important information about product profile. Additionally, and as highlighted by our study results, these PRO are seen by EMA as data supporting efficacy and safety, so claims may be supported by PRO as secondary endpoints, exploratory endpoints or both (6, 11, 20). Despite this, there is only a small number of claims granted, which may be attributed to the time and budget constraints of a clinical trial, potentially influencing sponsors to invest mostly in the primary endpoints (6).

The extent and reasons for excluding PRO from labeling are still unclear, however, misalignment among regulatory agencies and lack of interest from sponsors to invest in PRO data are the key drivers for the poor inclusion of patients' perspective on oncology medicinal products approval decisions. The selection of PROM should be discussed at an early dialogue with regulatory agencies to ensure they are acceptable for the target population, purpose and design of the study, but also for regulatory purposes.

### Guidance evolution and future directions

A recent review identified seven documents from the FDA, EMA and scientific consortia providing orientations regarding PRO use in clinical trials covering PROM selection, PRO protocols, PRO data analysis, interpretation and reporting (54, 55). Despite the continuous development of such guidelines and recommendations, the use of PRO has remained challenging and relatively constant throughout the years. Hence, additional research is needed to assess the consistency and disparities amongst its utility, in order to understand whether it is due to suboptimal compliance or whether the recommendations are unclear and/or difficult to interpret. A concern requiring attention from all stakeholders is the development and implementation of adequate protocols to collect and analyze PRO data. A recent study on oncology trials performed by Kyte et al. (56) identified that PRO data from 49,568 participants was not published due to suboptimal PRO protocol content Failure to report PRO findings is common, meaning that this information may not be accessible for the benefit of patients, clinicians and regulators. As an improved PRO protocol content is associated with more complete reporting, efforts are needed to improve guidance on the subject and consequently increase compliance by sponsors.

### Strengths and limitations

It is important to also acknowledge the limitations of our study. Firstly, this study included only clinical trials of EPARs remit, which were mainly phase III pivotal trials. Consequently, no comprehensive interpretations can be drawn about the PRO measures used in the development of new oncology medicines. Despite this, phase I and II clinical trials typically do not include PRO or do so as a component of PRO development and testing. Secondly, as this review only examined new oncology indications approved by EMA between 2017 and 2020, PRO use and PRO labeling claims on cancer medicines, cannot be fully characterized. Finally, it was inferred that when including a PRO in a clinical trial, a PRO label claim was intended, which could have biased our results interpretation.

Despite the limitations described above, this study has several strengths that also need to be acknowledged. Compared with previous reviews of PRO label claims in oncology medicinal products, this review provides a more comprehensive overview of how PRO measures have been used to support regulatory process and how they have been reviewed by EMA, allowing for a more detailed analysis of approval process of PRO label claims. By including the review of EPARs and SmPCs, this study enabled the identification of potential causes for PRO claims not being granted. Such insights provide an important perspective on the future challenges of using PROs in oncology clinical trials field.

## Conclusion

Despite growing recognition on the value of PRO data for the development of improved cancer therapies, EMA granted PRO claims to only 22 (17.2%) of the oncology indications approved between 2017 and 2020. 100 (78.1%) oncology indications included PROM in the confirmatory study(ies) supporting the submission, and PROM selection relied on the generic measure EQ-5D (29.2%) and the disease-specific measures EORTC (41.3%), and FACIT (17.1%). This study allowed to draw assumptions regarding the suboptimal use of PRO data in oncology clinical trials. Several key concerns were identified regarding PRO protocols including rationale, data collection, training, management and analysis, influence of study design, missing data and PROM selection. Additionally, we prompted the discussion about possible solutions to decrease these flaws, contributing for an optimal patient-centered healthcare system. To ensure PRO claims are granted by regulators, sponsors should consider, at a minimum, the use of a PROM that assesses concepts of the target population and disease for which it is intended, treatment-related symptoms, impact on functioning and HRQoL. While PRO implementation remains challenging, we believe that there is added value benefits in their use, namely by monitoring symptoms in individual patients, contributing to share decision-making processes, supporting health economic decisions, and ultimately enhancing healthcare systems.

## Author contributions

MT drafted the manuscript. All authors provided substantial input in the study design, provided a critical revision of the manuscript, read, and approved the final manuscript.

## Conflict of interest

The authors declare that the research was conducted in the absence of any commercial or financial relationships that could be construed as a potential conflict of interest.

## Publisher's note

All claims expressed in this article are solely those of the authors and do not necessarily represent those of their affiliated organizations, or those of the publisher, the editors and the reviewers. Any product that may be evaluated in this article, or claim that may be made by its manufacturer, is not guaranteed or endorsed by the publisher.
