# Supplementary material for: A review of patient-reported outcomes used for regulatory approval of oncology medicinal products in the European Union between 2017 and 2020
Source: Front Med (Lausanne). 2022 Aug 12;9:968272. doi: 10.3389/fmed.2022.968272 (PMC9411861; doi:10.3389/fmed.2022.968272)
Supplement: Supplementary file 1 [file Data_Sheet_1.docx]

Supplementary Material

**Annex 1 - List of medicines included**

| International Non-Proprietary Name (INN) | Therapeutic Indication | Approval Year | Line Extensions |
| --- | --- | --- | --- |
| Abemaciclib | Breast cancer | 2018 |  |
| Abiraterone acetate | Metastatic hormone sensitive prostate cancer (mHSPC) | 2017 | x |
| Acalabrutinib | Chronic lymphocytic leukaemia (CLL) | 2020 |  |
|  | Chronic lymphocytic leukaemia (CLL) | 2020 |  |
| Alectinib | Non-small cell lung cancer (NSCLC) | 2017 | x |
| Alpelisib | Breast cancer | 2020 |  |
| Apalutamide | Prostate cancer | 2019 | x |
|  | Prostate cancer | 2020 |  |
| Atezolizumab | Urothelial carcinoma (UC) | 2017 |  |
|  | Non-small cell lung cancer (NSCLC) | 2017 | x |
|  | Non-small cell lung cancer (NSCLC) | 2019 | x |
|  | Triple-negative breast cancer (TNBC) | 2019 |  |
|  | Hepatocellular carcinoma (HCC) | 2020 |  |
| Autologous anti-CD19-transduced CD3+ cells | Mantle cell lymphoma (MCL) | 2020 |  |
| Avapritinib | Gastrointestinal stromal tumours (GIST) | 2020 |  |
| Avelumab | Merkel cell carcinoma (MCC) | 2017 |  |
|  | Renal cell carcinoma (RCC) | 2019 |  |
| Axicabtagene ciloleucel | Diffuse large B-cell lymphoma (DLBCL) + primary mediastinal large B-cell lymphoma (PMBCL) | 2018 |  |
| Belantamab mafodotin | Multiple myeloma (MM) | 2020 |  |
| Bevacizumab | Ovarian, fallopian, primary peritoneal | 2017 | x |
| Binimetinib | Melanoma | 2018 |  |
| Blinatumomab | Acute lymphoblastic/lymphocytic leukaemia (ALL) | 2018 | x |
|  | Acute lymphoblastic/lymphocytic leukaemia (ALL) | 2019 |  |
|  | Acute lymphoblastic/lymphocytic leukaemia (ALL) | 2020 | x |
| Bosutinib | Chronic myelogenous leukaemia (CML) | 2018 | x |
| Brentuximab vedotin | T-cell lymphoma | 2017 |  |
|  | Hodgkin lymphoma | 2019 |  |
|  | T-cell lymphoma | 2020 |  |
| Brigatinib | Non-small cell lung cancer (NSCLC) | 2018 |  |
|  | Non-small cell lung cancer (NSCLC) | 2020 |  |
| Cabozantinib | Renal cell carcinoma (RCC) | 2018 | x |
|  | Hepatocellular carcinoma (HCC) | 2018 |  |
| Carfilzomib | Multiple myeloma (MM) | 2020 | x |
| Cemiplimab | Metastatic or locally advanced cutaneous squamous cell carcinoma (mCSCC or laCSCC) | 2019 |  |
| Ceritinib | Non-small cell lung cancer (NSCLC) | 2017 |  |
| Dabrafenib | Non-small cell lung cancer (NSCLC) | 2017 |  |
|  | Melanoma | 2018 | x |
| Daratumumab | Multiple myeloma (MM) | 2017 | x |
|  | Multiple myeloma (MM) | 2018 | x |
|  | Multiple myeloma (MM) | 2019 | x |
|  | Multiple myeloma (MM) | 2020 | x |
| Dacomitinib | Non-small cell lung cancer (NSCLC) | 2019 |  |
| Darolutamide | Non-metastatic castration resistant prostate cancer (nmCRPC) | 2020 |  |
| Dasatinib | Acute lymphoblastic/lymphocytic leukaemia (ALL) | 2019 | x |
| Daunorubicin / cytarabine | Acute myeloid/myeloblastic leukaemia (AML) | 2018 |  |
| Dinutuximab beta | Neuroblastoma | 2017 |  |
| Docetaxel | Prostate cancer | 2019 | x |
| Durvalumab | Non-small cell lung cancer (NSCLC) | 2018 |  |
|  | Non-small cell lung cancer (NSCLC) | 2020 | x |
| Elotuzumab | Multiple myeloma (MM) | 2019 | x |
| Encorafenib | Melanoma | 2018 |  |
|  | Metastatic colorectal cancer (CRC) | 2020 |  |
| Entrectinib | NSCLC + solid cancer | 2020 |  |
| Enzalutamide | Prostate cancer | 2018 |  |
| Fulvestrant | Breast cancer | 2017 |  |
|  | Breast cancer | 2017 | x |
| Gemtuzumab ozogamicin | Acute myeloid/myeloblastic leukaemia (AML) | 2018 |  |
| Glasdegib | Acute myeloid/myeloblastic leukaemia (AML) | 2020 |  |
| Gilteritinib | Acute myeloid/myeloblastic leukaemia (AML) | 2019 |  |
| Ibrutinib | Chronic lymphocytic leukaemia (CLL) | 2019 | x |
|  | Waldenström’s macroglobulinaemia (WM) | 2019 | x |
|  | Chronic lymphocytic leukaemia (CLL) | 2020 | x |
| Ipilimumab | Melanoma | 2018 | x |
|  | Melanoma | 2018 | x |
|  | Renal cell carcinoma (RCC) | 2019 |  |
|  | Non-small cell lung cancer (NSCLC) | 2020 |  |
| Isatuximab | Multiple myeloma (MM) | 2020 |  |
| Larotrectinib | Abdominal neoplasms | 2019 |  |
| Lenalidomide | Multiple myeloma (MM) | 2017 | x |
|  | Multiple myeloma (MM) | 2019 | x |
|  | Follicular lymphoma (FL) | 2019 |  |
| Lenvatinib | Hepatocellular carcinoma (HCC) | 20178 |  |
| Lorlatinib | Non-small cell lung cancer (NSCLC) | 2019 |  |
| Lutetium (177Lu) oxodotreotide | Gastroenteropancreatic neuroendocrine tumours (GEP NETs) | 2017 |  |
| Midostaurin | Aggressive systemic mastocytosis (ASM), systemic mastocytosis with associated haematological neoplasm (SM AHN), or MCL | 2017 |  |
|  | Acute myeloid/myeloblastic leukaemia (AML) | 2017 |  |
| Mogamulizumab | Mycosis fungoides (MF) or Sézary syndrome (SS) | 2018 |  |
| Neratinib | Breast Cancer | 2018 |  |
| Nilotinib | Chronic myelogenous leukaemia (CML) | 2017 | x |
| Niraparib | Ovarian, fallopian, primary peritoneal | 2017 |  |
|  | Ovarian, fallopian, primary peritoneal | 2020 | x |
| Nivolumab | Head and neck squamous cell carcinoma (HNSCC) | 2017 |  |
|  | Urothelial carcinoma (UC) | 2017 |  |
|  | Adjuvant treatment of melanoma | 2018 |  |
|  | Renal cell carcinoma (RCC) | 2019 | x |
|  | Non-small cell lung cancer (NSCLC) | 2020 | x |
|  | Oesophageal squamous cell carcinoma (OSCC) | 2020 |  |
| Obinutuzumab | Follicular lymphoma (FL) | 2017 |  |
| Olaparib | Breast cancer | 2019 |  |
|  | Ovarian, fallopian, primary peritoneal | 2019 | x |
|  | Ovarian, fallopian, primary peritoneal | 2020 | x |
|  | Prostate cancer | 2020 |  |
|  | Adenocarcinoma of the pancreas | 2020 |  |
| Osimertinib | Non-small cell lung cancer (NSCLC) | 2018 | x |
| Paclitaxel | Ovarian, fallopian, primary peritoneal | 2018 |  |
| Padeliporfin | Prostate cancer | 2017 |  |
| Pembrolizumab | Classical hodgkin lymphoma (CHL) | 2017 |  |
|  | Urothelial carcinoma (UC) | 2017 |  |
|  | Melanoma | 2018 |  |
|  | Non-small cell lung cancer (NSCLC) | 2019 |  |
|  | Renal cell carcinoma (RCC) | 2019 |  |
|  | Head and neck squamous cell carcinoma (HNSCC) | 2019 |  |
| Pertuzumab | Breast cancer | 2018 | x |
| Pertuzumab / trastuzumab | Breast cancer | 2020 |  |
| Polatuzumab vedotin | Diffuse large B-cell lymphoma (DLBCL) | 2020 |  |
| Pomalidomide | Multiple myeloma (MM) | 2019 | x |
| Ramucirumab | Hepatocellular carcinoma (HCC) | 2019 |  |
|  | Non-small cell lung cancer (NSCLC) | 2019 | x |
| Regorafenib | Hepatocellular carcinoma (HCC) | 2017 |  |
| Ribociclib | Early breast cancer (EBC) + metastatic breast cancer (MBC) | 2017 |  |
| Rituximab | B-cell lymphoma | 2020 |  |
| Rucaparib | Ovarian, fallopian, primary peritoneal | 2018 |  |
|  | Ovarian, fallopian, primary peritoneal | 2019 |  |
| Talazoparib | Breast Cancer | 2019 |  |
| Trametinib | Non-small cell lung cancer (NSCLC) | 2017 |  |
|  | Melanoma | 2018 | x |
| Tisagenlecleucel | ALL | 2018 |  |
|  | Diffuse large B-cell lymphoma (DLBCL) | 2018 |  |
| Tivozanib hydrochloride monohydrate | Renal cell carcinoma (RCC) | 2017 |  |
| Trastuzumab emtansine | Early breast cancer (EBC) | 2019 | x |
| Trifluridine / tipiracil | Gastric cancer | 2019 |  |
| Venetoclax | Chronic lymphocytic leukaemia (CLL) | 2018 | x |
|  | Chronic lymphocytic leukaemia (CLL) | 2020 | x |
| 5-aminolevulinic acid hydrochloride | Basal cell carcinoma | 2017 | x |

**Annex 2 – Patient-reported-outcome measure referenced in the review of the 100 indications with patient-reported-outcomes data**

**Figure 1 - Generic patient-reported outcome measures used (n= 100 indications with PRO)**

Figure 2 - Disease-specific patient-reported outcome measures used

**Annex 3: Summary of product of characteristics claims**

**Acute Lymphoblastic/lymphocytic Leukemia**

**Tisagenlecleucel**

“Health-related quality of life (HRQoL) was evaluated by PedsQL and EQ-5D questionnaires completed by patients aged 8 years and above (n=61). Among patients responding (n=51), the mean (SD) change from baseline in the PedsQL total score was 13.1 (13.45) at month 3, 15.4 (16.81) at month 6 and 25.0 (19.09) at month 12, and the mean (SD) change from baseline in the EQ-5D VAS score was 16.0 (16.45) at month 3, 15.3 (18.33) at month 6 and 21.7 (17.14) at month 12, indicating overall clinically meaningful improvement in HRQoL following Kymriah infusion.”

**Breast Neoplasms**

**Abemaciclib**

“Verzenios plus fulvestrant prolonged progression-free survival with neither a clinically meaningful or significant detriment to health-related quality of life.”

**Fulvestrant**

**“**Patient-reported symptoms were assessed using the European Organization for Research and Treatment of Cancer (EORTC) quality of life questionnaire (QLQ)-C30 and its Breast Cancer Module (EORTC QLQ-BR23). A total of 335 patients in the Faslodex plus albociclib arm and 166 patients in the Faslodex plus placebo arm completed the questionnaire at baseline and at least 1 post-baseline visit.”

**Pertuzumab**

“Secondary endpoints included the assessment of patient-reported global health status, role and physical function, and treatment symptoms using the EORTC QLQ-C30 and EORTC QLQ-BR23 questionnaires. In the analyses of patient-reported outcomes, a 10-point difference was considered clinically meaningful. Patients’ physical function, global health status and diarrhoea scores showed a clinically meaningful change during chemotherapy in both treatment arms. The mean decrease from baseline at that time for physical function was -10.7 (95% CI-11.4, -10.0) in the Perjeta arm and -10.6 (95% CI -11.4, -9.9) in the placebo arm; global health status was -11.2 (95% CI -12.2, -10.2) in the Perjeta arm and -10.2 (95% CI -11.1,-9.2) in the placebo arm. Change in diarrhoea symptoms increased to +22.3 (95% CI 21.0, 23.6) in the Perjeta arm versus +9.2 (95% CI 8.2, 10.2) in the placebo arm. Thereafter in both arms physical function and global health status scores returned to baseline levels during targeted treatment. Diarrhoea symptoms returned to baseline after HER2 therapy in the Perjetaarm. The addition of Perjeta to trastuzumab plus chemotherapy did not affect patients’ overall role function over the course of the study.”

**Ribociclib**

“The global health status/QoL data showed no relevant difference between the Kisqali plus letrozole arm and the placebo plus letrozole arm.”

**Follicular lymphoma**

**Obinutuzumab**

**“**Patient Reported Outcomes Based on the FACT-Lym questionnaire collected during treatment and follow-up phases, patients in both treatment arms experienced clinically meaningful improvements in lymphoma-related symptoms as defined by a ≥ 3 point increase from baseline in the Lymphoma subscale, a ≥ 6 point increase from baseline in the FACT Lym TOI and a ≥ 7 point increase from baseline in the FACT Lym Total score. EQ-5D utility scores were similar at baseline, during treatment and follow-up. No meaningful differences were seen between the arms in HRQOL or health status measures. Due to the open label design the patient reported outcomes should be interpreted with caution.”

**Hepatocellular carcinoma**

**Cabozantinib**

“Non-disease specific quality of life (QoL) was assessed using the EuroQoL EQ-5D-5L. A negative effect of CABOMETYX versus placebo on the EQ-5D utility index score was observed during the first weeks of treatment. Only limited QoL data are available after this period*.”*

**Hodgkin lymphoma**

**Brentuximab vedotin**

“The European Organization for Research and Treatment of Cancer Quality of Life 30-Item Questionnaire (EORTC-QLQ-C30) showed no clinically meaningful difference between the two arms in both the ITT and Stage IV population.”

**Melanoma**

**Binimetinib**

**“**The Functional Assessment of Cancer Therapy-Melanoma (FACT-M), the European Organisation for Research and Treatment of Cancer’s core quality of life questionnaire (EORTC QLQ-C30) and the EuroQoL-5 Dimension-5 Level examination (EQ-5D-5L) were used to explore patient-reported outcomes (PRO) measures of health-related Quality of Life, functioning, melanoma symptoms, and treatment-related adverse reaction. A definitive 10% deterioration in FACT-M and in EORTC QLQ-C30 was significantly delayed in patients treated with Combo 450 relative to other treatments. The median time to definitive 10 % deterioration in the FACT-M score was not reached in the Combo 450 arm and was 22.1 months (95 % CI: 15.2, NE) in the vemurafenib arm with a HR for the difference of 0.46 (95 % CI: 0.29, 0.72). An analysis of time to definitive 10 % deterioration in EORTC QLQ-C30 score provided with similar results. Patients receiving Combo 450 reported no change or a slight improvement in the mean change from baseline EQ-5D-5L index score at all visits, whilst patients receiving vemurafenib or encorafenib reported decreases at all visits (with statistical significant differences). An evaluation of change over time in score yielded the same trend for EORTC QLQ-C30 and at all visit for FACT-M.”

**Encorafenib**

**“**Quality of Life (QoL) (cut-off date: 19 May 2016) The Functional Assessment of Cancer Therapy-Melanoma (FACT-M), the European Organisation for Research and Treatment of Cancer’s core quality of life questionnaire (EORTC QLQ-C30) and the EuroQoL-5 Dimension-5 Level examination (EQ-5D-5L) were used to explore patient-reported outcomes (PRO) measures of health-related Quality of Life, functioning, melanoma symptoms, and treatment-related adverse reactions. A definitive 10% deterioration in FACT-M and in EORTC QLQC30 was significantly delayed in patients treated with Combo 450 relative to other treatments. The median time to definitive 10% deterioration in the FACT-M score was not reached in the Combo 450 arm and was 22.1 months (95% CI: 15.2, NE) in the vemurafenib arm with a HR for the difference of 0.46 (95% CI: 0.29, 0.72). An analysis of time to definitive 10% deterioration in EORTC QLQ-C30 score provided with similar results. Patients receiving Combo 450 reported no change or a slight improvement in the mean change from baseline EQ-5D-5L index score at all visits, whilst patients receiving vemurafenib or encorafenib reported decreases at all visits (with statistical significant differences). An evaluation of change over time in score yielded the same trend for EORTC QLQ-C30 and at all visit for FACT-M.”

**Nivolumab**

“Quality of life (QoL) with nivolumab remained stable and close to baseline values during treatment, as assessed by valid and reliable scales like the European Organization for Research and Treatment of Cancer (EORTC) QLQ-C30 and the EQ-5D utility index and visual analog scale (VAS)”

**Non-Small-Cell Lung Carcinoma**

**Atezolizumab**

“Prolonged time to deterioration of patient-reported pain in chest as measured by the EORTC QLQ-LC13 was observed with atezolizumab compared to docetaxel (HR of 0.71, 95% CI: 0.49, 1.05; median not reached in either arm). The time to deterioration in other lung cancer symptoms (i.e. cough, dyspnoea, and arm/shoulder pain) as measured by the EORTC QLQ-LC13 was similar between atezolizumab and docetaxel. These results should be interpreted with caution due to the open-label design of the study.”

**Atezolizumab**

“The time to deterioration (a sustained ≥ 10-point decline from baseline score) of patient-reported global health status/health-related quality of life as measured by the EORTC QLQ-C30 was similar in each treatment group indicating that all patients maintained their baseline HRQoL for a comparable duration of time.”

**Ceritinib**

“Patient reported outcome questionnaires (Lung cancer symptom scale [LCSS], EORTC-QLQ-C30 [C30], EORTC QLQ-LC13 [LC13] and EQ-5D-5L) were completed by 80% or more of patients in the ceritinib and chemotherapy arms for all questionnaires at most of the time-points during the course of the study. Ceritinib significantly prolonged time to deterioration for the pre-specified lung cancer specific symptoms of interest of cough, pain and dyspnoea (composite endpoint LCSS: HR=0.61, 95% CI:0.41, 0.90, median Time to Deterioration [TTD] NE [95% CI: 20.9, NE] in the ceritinib arm versus 18.4 months [13.9, NE] in the chemotherapy arm; LC13: HR=0.48, 95% CI: 0.34, 0.69, median TTD 23.6 months [95% CI: 20.7, NE] in the ceritinib arm versus 12.6 months [95% CI: 8.9, 14.9] in the chemotherapy arm).

Patients receiving ceritinib showed significant improvements over chemotherapy in general Quality of Life and global Health Status measures (LCSS [p<0.001], QLQ-C30, [p<0.001] and EQ-5D-5L index [p<0.001])."

**Durvalumab**

**“**Patient-reported symptoms, function and health-related quality of life (HRQoL) were collected using the EORTC QLQ-C30 and its lung cancer module (EORTC QLQ-LC13). The LC13 and C30 were assessed at baseline, every 4 weeks for the first 8 weeks, followed by every 8 weeks until completion of the treatment period or discontinuation of IMFINZI due to toxicity or disease progression. Compliance was similar between the IMFINZI and placebo treatment groups (83% vs. 85.1% overall of evaluable forms completed). At baseline, no differences in patient-reported symptoms, function and HRQoL were observed between IMFINZI and placebo groups. Throughout the duration of the study to Week 48, there was no clinically meaningful difference between IMFINZI and placebo groups in symptoms, functioning and HRQoL (as assessed by a difference of greater than or equal to 10 points).”

**Osimertinib**

“Patient Reported Outcomes

Patient-reported symptoms (PRO) and health-related quality of life (HRQL) were electronically collected using the EORTC QLQ-C30 and its lung cancer module (EORTC QLQ-LC13). The LC13 was initially administered once a week for the first 6 weeks, then every 3 weeks before and after progression. The C30 was assessed every 6 weeks before and after progression. At baseline, no differences in patient reported symptoms, function or HRQL were observed between TAGRISSO and EGFR TKI comparator (gefitinib or erlotinib) arms. Compliance over the first 9 months was generally high (≥70%) and similar in both arms.

Key lung cancer symptoms analysis

Data collected from baseline up to month 9 showed similar improvements in TAGRISSO and EGFR TKI comparator groups for the five pre-specified primary PRO symptoms (cough, dyspnoea, chest pain, fatigue, and appetite loss) with improvement in cough reaching the established clinically relevant cut-off. Up to month 9 there were no clinically meaningful differences in patient-reported symptoms between TAGRISSO and EGFR TKI comparator groups (as assessed by a difference of ≥10 points).

HRQL and physical functioning improvement analysis

Both groups reported similar improvements in most functioning domains and global health status/HRQL, indicating that patients’ overall health status improved. Up to month 9, there were no clinically meaningful differences between the TAGRISSO and EGFR TKI comparator groups in functioning or HRQL."

**Ovarian Neoplasms**

**Niraparib**

“Patient-reported outcome (PRO) data from validated survey tools (FOSI and EQ-5D) indicate that niraparib-treated patients reported no difference from placebo in measures associated with quality of life (QoL).”

**Pancreatic Neoplasms**

**Lutetium (177Lu) oxodotreotide**

“Health Related Quality of Life (HRQOL) was assessed using the European Organisation for Research and Treatment of Cancer Quality of Life Questionnaire (EORTC QLQ-C30) (generic instrument) and its neuroendocrine tumour module (EORTC QLQ-GI.NET-21). The results indicate an improvement in the overall global health-related quality of life up to week 84, for patients on Lutathera treatment as compared to patients on Octreotide LAR.”

**Prostatic Neoplasms**

**Apalutamide**

“There were no detrimental effects to overall health-related quality of life with the addition of Erleada to ADT and a small but not clinically meaningful difference in change from baseline in favor of Erleada observed in the analysis of the Functional Assessment of Cancer Therapy-Prostate (FACT-P) total score and subscales.”

**Padeliporfin**

“Effect on urinary morbidity (IPSS) and erectile function (IIEF) following TOOKAD-VTP As shown in Table 5, in PCM301 study, the International Prostate Symptoms Score (IPSS) showed, a moderate increase 7 days after the VTP procedure, in both the ITT population and in patients meeting the indication criteria. Those results were improved at Month 3 and back to baseline values at Month 6, with further improvement until Month 24. In the Active Surveillance arm, the IPSS score slightly worsened over time until Month 24.

As shown in Table 6, in the VTP arm of PCM301 study, erectile function domain scores of the 15-question International Index of Erectile Function (IIEF-15) questionnaire showed a marked decrease, 7 days after the VTP procedure followed by a subsequent improvement in the following months up to Month 24, in the ITT population and in patients meeting the indication criteria.”

**T-cell Lymphoma**

**Brentuximab vedotin**

“No meaningful differences in quality of life (assessed by the EuroQol five dimensions questionnaire [EQ-5D] and Functional Assessment of Cancer Therapy-General [FACT-G]) were observed between the treatment arms.”

**Urologic Neoplasms**

**Pembrolizumab**

“Patient-reported outcomes (PROs) were assessed using EORTC QLQ-C30. A prolonged time to deterioration in EORTC QLQ-C30 global health status/QoL was observed for patients treated with pembrolizumab compared to investigator’s choice chemotherapy (HR 0.70; 95% CI 0.55-0.90). Over 15 weeks of follow-up, patients treated with pembrolizumab had stable global health status/QoL, while those treated with investigator’s choice chemotherapy had a decline in global health status/QoL. These results should be interpreted in the context of the open-label study design and therefore taken cautiously.”
